# Cell-Free Expression of Unnatural Amino Acid Incorporated Aquaporin SS9 with Improved Separation Performance in Biomimetic Membranes

**DOI:** 10.1155/2018/3560894

**Published:** 2018-09-27

**Authors:** Peilian Wei, Bingjia Zhuang, Daoyong Yu, Aziza Sharipova, Jin Cai, Lei Huang, Jiazhang Lian, Zhinan Xu

**Affiliations:** ^1^School of Biological and Chemical Engineering, Zhejiang University of Science & Technology, Hangzhou 310023, China; ^2^College of Chemical and Biological Engineering, Zhejiang University, Hangzhou 310027, China; ^3^College of Chemical Engineering, China University of Petroleum (East China), Qingdao 266580, China

## Abstract

Aquaporins (AQPs) are widely applied in biomimetic membranes for water recycling and desalination. In this study, a novel aquaporin was isolated from* Photobacterium profundum *SS9 (AQP SS9), which showed high water permeability and potential for practical water purification applications. To improve the stability of the AQP SS9 embedded biomimetic membranes, a modified AQP SS9 was obtained by incorporation of an unnatural amino acid (*p*-propargyloxyphenylalanine,* p*Ppa) (P-AQP SS9)* in vitro *using a mutated* Methanocaldococcus jannaschii* tyrosyl-tRNA synthetase (TyrRS) and the cell-free expression system. The modified AQP SS9 can covalently link with phospholipids and hence significantly improve the stability of biomimetic membranes. The concentration of Mg^2+^ and fusion expression with signal peptides were evaluated to enhance the expression level of P-AQP SS9, resulting in a highest yield of 49 mg/L. The modified AQP SS9 was then reconstituted into DOPC liposomes and analyzed by a stopped-flow spectrophotometer. The obtained water permeability coefficient (P_f_) of 7.46×10^−4^ m/s was 5.7 times higher than that of proteoliposomes with the wild-type AQP SS9 (P_f_=1.31×10^−4^ m/s) and 12.1 times higher than that of the DOPC liposomes (P_f_=6.15×10^−5^m/s). This study demonstrates the development of a cell-free system for the expression of membrane proteins with much higher stability and the potential application of the modified aquaporins for water filtration.

## 1. Introduction

Aquaporins belong to a large family of water-channel proteins, and the so-called orthodox aquaporins (AQPs) possess highly defined nanoscale pores to allow water molecules to rapidly pass through while retaining the dissolved solutes effectively [1]. The incorporation of AQPs into the lipid bilayer could enhance the permeability of reconstituted bilayer by an order of magnitude, while the high retention of solutes was still maintained [2]. Such ideal separation properties of aquaporins have led to an intensive interest in synthesizing aquaporin based high-performance biomimetic membranes, especially for desalination applications [3, 4].

Considering the high osmotic pressure and salinity conditions in practical applications, AQPs from the barophilic bacteria could be suitable and promising candidates for the fabrication of biomimetic membranes.* Photobacterium profundum SS9* was isolated from Sulu Trough amphipod living in 2550 meters undersea [5, 6] and has been classified as a moderate barophilic bacterium [7]. Therefore, the aquaporin from* P. profundum SS9 *(AQP SS9) should have certain advantages in resisting high hydrostatic pressure, high salinity, and some other extreme conditions in water filtration and desalination applications.

The tyrosyl-tRNA synthetase (TyrRS) from* Methanocaldococcus jannaschii *and its corresponding tRNA (MjTyrRS/MjtRNA^Tyr^) have been found to be able to identify the amber codon (TAG) and transport tyrosine [8]. Further study [9] showed that the mutated MjTyrRS/MjtRNA^Tyr^ could transport certain unnatural amino acids to the TAG codon of a target gene, thereby enhancing the performance of protein, stabilizing protein structure, and even generating new functions. More than 90 unnatural amino acids have been site-specifically incorporated into a variety of proteins via the gene coding technology [8].

**Figure 1:**
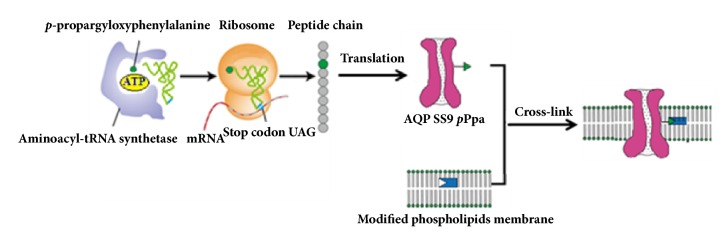
Schematic diagram of genetic incorporation of* p*Ppa into AQP SS9.

In this study, AQP SS9 was firstly phylogenetically characterized as an orthodox aquaporin. Then an unnatural amino acid was introduced into AQP SS9, which can link covalently with phospholipids to improve the stability of the biomimetic membranes. As shown in Figure 1, a stop codon TAG has been introduced into the expression vector pIVEX2.4c-AQP SS9 via site-directed mutagenesis, and the mutated* M. jannaschii* TyrRS could catalyze the aminoacylation of tRNA with the unnatural amino acid* p*-propargyloxyphenylalanine (*p*Ppa). Then,* p*Ppa incorporated AQP SS9 (P-AQP SS9) was efficiently expressed in the* Escherichia coli* derived cell-free system. Finally, P-AQP SS9 and AQP SS9 incorporated proteasomes were constructed and their separation performance (water permeability) was compared. The system with modified AQP SS9 showed much higher water permeability, demonstrating the potential for practical applications in wastewater treatment and seawater desalination.

## 2. Materials and Methods

### 2.1. Strains and Plasmids

*E. coli* DH5*α* was used for plasmid construction and* E. coli* BL21 (DE3) (Novangen, USA) as the host for making cell-free extract. pIVEX2.4c (Roche, Grenzacherstrasse, Switzerland) was used to construct cell-free expression vectors. pIVEX2.4c-AQP SS9, pETDuet-CK-T7, pUC-MjtRNA, p15a-Mj*p*PpaRS, and pIVEX2.4c-Mj*p*PpaRS were constructed in our previous studies.

### 2.2. Reagents

Restriction endonucleases, LA Taq DNA polymerase, and T4 DNA ligase were purchased from Takara Company (Dalian, China). All primers for PCR amplification were synthesized by Sangon Biotech (Shanghai, China) Co. Ltd. and listed in Table 1. The unnatural amino acid* p*-propargyloxyphenylalanine (*p*Ppa) was provided by Amatek Chemical Company. Western-blot reagents were purchased from Beyotime Biotechnology (Shanghai, China).

### 2.3. Vector Construction

The amber mutations were introduced into the sites of F35TAG and L39TAG of AQP SS9 using the primer set AQP SS9TAG-F/AQP SS9TAG-R with pIVEX2.4c-AqpSS9 as the template, resulting in the construction of pIVEX2.4c-AQP SS9TAG. As signal peptides (sp) were found to enhance the expression level of target proteins in cell-free system [10], six fusion expression plasmids including pIVEX2.4c-pelBsp-AQP SS9TAG, pIVEX2.4c-ompCsp-AQP SS9TAG, pIVEX2.4c-ompAsp-AQP SS9TAG, pIVEX2.4c-malEsp-AQP SS9TAG, pIVEX2.4c-dsbAsp-AQP SS9TAG, and pIVEX2.4c-phoAsp-AQP SS9TAG were constructed. The expression of the mutated AQP SS9 with different signal peptides including dsbAsp (GenBank ID BAL40432), malEsp (GenBank ID BAL40591), ompAsp (GenBank ID BAL38095), ompCsp (GenBank ID BAL39006), pelBsp (GenBank ID AAA24848), and phoAsp (GenBank ID BAL37591) was carried out according to the previously reported method [11]. The signal peptide sequences were predicted using the Signal Peptide Database (http://proline.bic.nus.edu.sg/spdb/index.html) (Box 1).

### 2.4. Expression and Purification of Proteins Using* E. coli* Derived Cell-Free Expression System

Two plasmids pUC-MjtRNA and p15a-Mj*p*PpaRS were cotransformed into* E. coli* BL21 (DE3), which was used for the preparation of cell-free extracts following a previously reported protocol [12]. The cell-free extracts were stored at -80°C for future use. The* E. coli* cell-free expression system was set up accordingly with minor modifications [11, 12]. The unnatural amino acid* p*Ppa was prepared as a stock solution of 50 mM and supplemented to the cell-free system at a final concentration of ~0.45 mM, unless specifically mentioned. 1% Brij58 was supplemented to the reaction mixture for soluble expression of the membrane protein. The cell-free reaction mixture was shaken at 400 rpm and 37°C (thermostat metal bath) for 4 h and then centrifuged at 12000 g for 10 min. The resulting supernatant was diluted 5 times with column buffer (20 mM Tris-HCl, pH 7.8, 300 mM NaCl, 10 mM imidazole, 0.05% Brij-78) and mixed with 1 mL Ni^2+^-NTA resin and then shaken at 4°C and 120 rpm for additional 1 h. After rinsed with 5 mL column buffer, the target protein was eluted with 1 mL wash buffer (MOPS-KOH, pH 7.5, 300 mM imidazole, 0.05% Brij-78).

### 2.5. Preparation of AQP SS9 and P-AQP SS9 Proteoliposomes

To prepare AQP derived proteoliposome, 100 *μ*g/mL AQP SS9 or P-AQP SS9, 4 mg/mL DOPC liposome and 0.25% Triton X-100 (m/v) [13] were mixed and shaken at 25°C, 120 rpm for 30 min. Then 0.2 g/mL SM-2 Bio-Beads (Sigma, St. Louis, MO, USA) were added to the mixture and shaken for additional 2 h. The reaction mixture was ultracentrifuged at 300000 g and 4°C for 15 min, and the precipitates were washed and resuspended with MOPS-KOH buffer (100 mM, pH 7.5) to get the AQP SS9 or P-AQP SS9 proteoliposomes.

### 2.6. Water Permeability Assay of Reconstituted P-AQP SS9 Proteoliposomes

The water permeability of DOPC liposome, AQP SS9 proteoliposome, and P-AQP SS9 proteoliposome was measured by light scattering at 436 nm, 500 V, and 25°C using a stopped-flow spectrophotometer (SFM-300, BioLogic) [14]. An equal volume of liposome or proteoliposome solution (100 *μ*g/mL) and hypertonic solution (100 mM MOPS-KOH, pH 7.5, 400 mM NaCl) was mixed and measured under the reported conditions [15].

## 3. Results

### 3.1. Phylogenetic Analysis of AQP SS9

**Figure 2:**
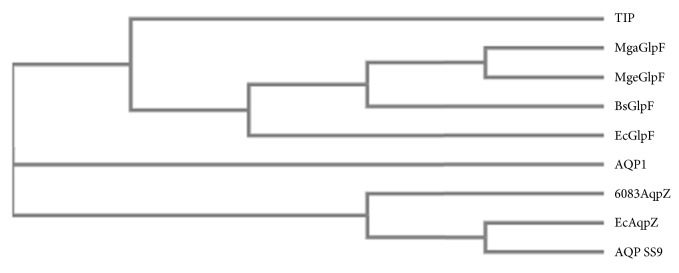
Phylogenetic analysis of AQP SS9. The amino acid sequences of aquaporin homologs were aligned and the phylogenetic tree was constructed. TIP, a plant aquaporin (NCBI Accession Number P25818.1); MgaGlpF,* Mycoplasma gallisepticum* aquaglyceroporin (NCBI Accession Number ADC30962.1); MgeGlpF,* Mycoplasma genitalium* aquaglyceroporin (NCBI Accession Number AAC71249.1); BsGlpF,* Bacillus subtilis* aquaglyceroporin (NCBI Accession Number AOT51422.1); EcGlpF,* E. coli* aquaglyceroporin (NCBI Accession Number NP_418362.1); AQP1, an animal aquaporin (NCBI Accession Number P29972.3); 6083AqpZ,* Synechocystis *sp. PCC6803 aquaporin Z (NCBI Accession Number P73809.1); EcAqpZ,* E. coli* aquaporin Z (NCBI Accession Number NP_415396.1).

Due to the lack of experimental data, a phylogenetic analysis was performed to study the evolutionary origins and the potential solute transport properties [16]. Some known bacterial orthodox aquaporins and aquaglyceroporins, as well as an animal (AQP1) and a plant (TIP) aquaporin, were aligned using Clustal Omega [17] and included in the phylogenetic tree. As shown in Figure 2, AQP SS9 showed the highest homology with the* E. coli* AqpZ [18], which has been characterized as an orthodox aquaporin and was not included in the aquaglyceroporin branch of the phylogenetic tree. Therefore, based on the phylogenetic analysis, AQP SS9 belongs to the orthodox aquaporin subfamily of water-channel proteins, which should transport water molecules efficiently and specifically.

### 3.2. Design of Unnatural Amino Acid Incorporation in AQP SS9

As the crystal structure of AQP SS9 has not been reported yet, the* E. coli* AqpZ with similar structure and high homology was selected for evaluation. Based on multiple sequence alignment (MSA) and analysis of the trans-membrane region of AqpZ by TMHMM, as well as the polarity of unnatural amino acids, F35 and L39 of AQP SS9 were chosen as the optimal sites for unnatural amino acid incorporation.

### 3.3. Optimization of the Expression of* p*Ppa Incorporated P-AQP SS9

**Figure 3:**
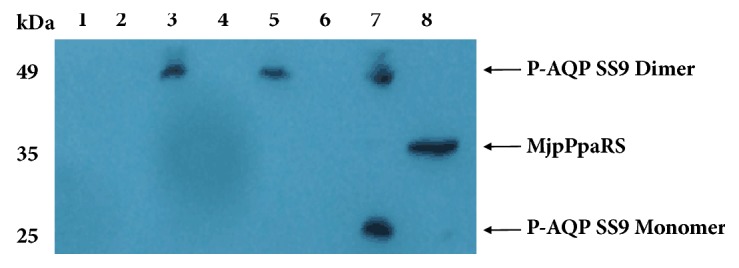
Western-blot analysis of* p*Ppa incorporated AQP SS9. Lane 1, pellet without* p*Ppa; Lane 2, supernatant without* p*Ppa; Lane 3, pellet with 2 *μ*L* p*Ppa; Lane 4, supernatant with 2 *μ*L* p*Ppa; Lane 5, pellet with 4 *μ*L* p*Ppa; Lane 6, supernatant with 4 *μ*L* p*Ppa; Lane 7, pellet with* p*Ppa and pIVEX2.4c-Mj*p*PpaRS; Lane 8, supernatant with* p*Ppa and pIVEX2.4c-Mj*p*PpaRS.

The pIVEX2.4c-AqpSS9TAG carrying amber mutations was expressed in* E. coli* cell-free expression system under different conditions. P-AQP SS9 expression could not be detected neither in the soluble nor in the insoluble fractions without the addition of* p*Ppa (Figure 3, Lane 1 and Lane 2), while the supplementation of 2 *μ*L* p*Ppa (~0.45 mM) led to the expression of P-AQP SS9 (Figure 3, Lane 3 and Lane 4). These results indicated that Mj*p*PpaRS/MjtRNA in the cell-free expression system could recognize the stop codon TAG and specifically incorporate* p*Ppa into AQP SS9. Since further increment of* p*Ppa up to 4 *μ*L could not improve the amount of the recombinant protein (Figure 3, Lane 5 and Lane 6), 2 *μ*L* p*Ppa was set as the optimal amount for subsequent studies. Although Mj*p*PpaRS has already been overexpressed in the cell-free extracts, supplementation of pIVEX2.4c-Mj*p*PpaRS further increased the production of P-AQP SS9 (Figure 3, Lane 7 and Lane 8), indicating that the amount of Mj*p*PpaRS was the bottleneck for efficient incorporation of* p*Ppa into the recombinant protein. Notably, in agreement with previous studies, all the membrane proteins were expressed as the insoluble form in the cell-free system (Figure 3). To enable soluble expression of P-AQP SS9, 1% Brij58 was supplemented into the cell-free system in the following studies.

**Figure 4:**
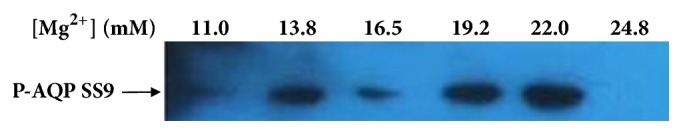
Effects of varied concentration of Mg^2+^ on AQP SS9 expression in cell-free system.

In addition, the effect of Mg^2+^ on the expression of P-AQP SS9 was also investigated. Varied concentration of Mg^2+^ (11.0, 13.8, 16.5, 19.2, 22.0, and 24.8 mM) was added to the cell-free expression system. Western-blot analysis showed that the highest expression level of P-AQP SS9 was achieved with 22.0 mM of Mg^2+^, while very low level of protein expression was observed when Mg^2+^ concentration was beyond the range from 11.0 mM to 22.0 mM (Figure 4).

### 3.4. Fusion Expression of P-AQP SS9 with Different Signal Peptides

**Figure 5:**
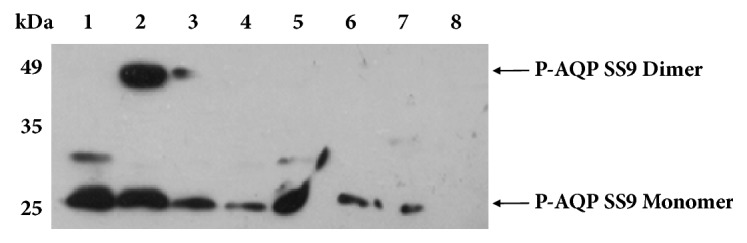
Effect of fusion expression of P-AQP SS9 with different signal peptides. Lane 1, pIVEX2.4c-AQP SS9TAG; Lane 2, pIVEX2.4c-dsbAsp-AQP SS9TAG; Lane 3, pIVEX2.4c-malEsp-AQP SS9TAG; Lane 4, pIVEX2.4c-ompAsp-AQP SS9TAG; Lane 5, pIVEX2.4c-ompCsp-AQP SS9TAG; Lane 6, pIVEX2.4c-pelBsp-AQP SS9TAG; Lane 7, pIVEX2.4c-phoAsp-AQP SS9TAG; Lane 8, negative control without the supplementation of any AQP SS9 expression plasmid.

The fusion of signal peptide at the N-terminus has been found to increase the expression level of recombinant proteins in cell-free system [11]. Therefore, multiple signal peptides including dsbAsp, malEsp, ompAsp, ompCsp, pelBsp, and phoAsp were chosen to further enhance the expression of P-AQP SS9. Among six signal peptides tested, dsbAsp enhanced the expression of P-AQP SS9 up to 49 mg/L, which was two times higher than that without any signal peptide (Figure 5). Interestingly, signal peptides pelBsp and phoAsp decreased the expression level of P-AQP SS9 to 11 mg/L.

### 3.5. Water Permeability of P-AQP SS9 Reconstituted Proteoliposomes

**Figure 6:**
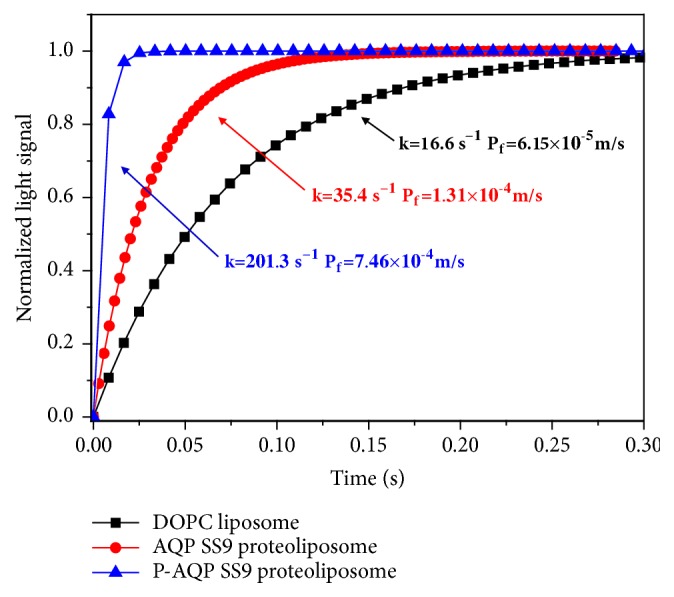
Water permeability of AQP SS9 and P-AQP SS9 proteoliposomes by stopped-flow spectrometer.

The two types of proteoliposomes with AQP SS9 and P-AQP SS9 were individually prepared and analyzed for its water permeability. The P_f_ of P-AQP SS9 proteoliposomes, AQP SS9 proteoliposomes, and control empty liposomes were 7.46×10^−4^ m/s, 1.31×10^−4^ m/s, and 6.15×10^−5^ m/s, respectively (Figure 6). The water permeable rate of P-AQP SS9 proteoliposomes was 5.7 times higher than that of AQP SS9 proteoliposomes, both of which were much higher than that of the empty liposomes. Thus, the modified P-AQP SS9 presented better water filtration activity compared with the wild-type AQP SS9.

## 4. Discussion

The AQPs incorporated biomimetic membranes have demonstrated better water permeability and salt rejection ability [19], but the low strength and poor integrity of the membranes are the biggest challenges for practical applications. To solve these defects, we have successfully incorporated* p*Ppa into AQP SS9 to achieve the covalent-link between the membrane proteins and the phospholipids bilayer. In this case, the strength and integrity of AQP embedded biomimetic membrane could be enhanced.

Considering the impact of unnatural amino acids on the 3D structure and water permeability of AQPs as well as the cross linking efficiency between AQPs and phospholipids, the type of unnatural amino acid and its incorporation sites in AQP SS9 should be rationally designed. Based on the modeling studies of AqpZ, which is highly homologous to AQP SS9, the active sites and phospholipids-linking regions of AQP SS9 were avoided for amino acid substitution, and finally F35TAG and L39TAG were selected as the mutation sites. On the other hand, since the covalent cross-link of acetylene group and phospholipids may improve the protein stability in the membrane,* p*Ppa was chosen as the target unnatural amino acid to be incorporated into AQP SS9.

To achieve our engineering goal, the* M. jannaschii *TyrRS mutant constructed in our previous studies was used for the synthesis of corresponding aminoacyl-tRNA, and the stop codon TAG was specifically introduced into the AQP SS9 gene by site-directed mutagenesis. Then* E. coli* cell-free expression system containing the engineered TyrRS could specifically incorporate* p*Ppa into the F35TAG and L39TAG sites of AQP SS9. The expression level of P-AQP SS9 in cell-free system was further improved through the optimization of the Mg^2+^ concentration (22.0 mM) and the fusion of signal peptide (dsbAsp), resulting in the production of 49 mg/L P-AQP SS9.

To characterize the obtained P-AQP SS9 and explore its possible applications, P-AQP SS9 was reconstituted into liposomes and biomimetic membranes. The water permeability factor (P_f_) of P-AQP SS9 liposomes was 7.46×10^−4^ m/s, 5.7 and 12.1 times higher than that of natural AQP SS9 and DOPC liposomes, respectively.

## 5. Conclusion

In this study, the* M. jannaschii *TyrRS mutant was used to incorporate the unnatural amino acid* p*Ppa into AQP SS9 (P-AQP SS9) via the* E. coli* cell-free expression system. The expression level of P-AQP SS9 in cell-free system was further improved by optimizing Mg^2+^ concentration and fusing with a signal peptide. Subsequent analyses showed that the water permeability of P-AQP SS9 had been significantly improved compared with that of AQP SS9.

## Figures and Tables

**Box 1:**
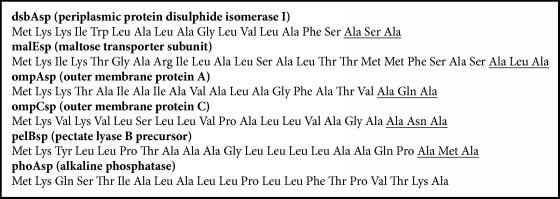
Signal peptides used in this study to enhance the expression of AQP SS9 in cell-free system. The peptidase cleavage sites Ala-X-Ala were underlined.

**Table 1:** Primers used in this study.

Primers	Sequence
AQP SS9TAG-F	5′-gtggctgaattcattggcacatAGtggttagtatAGgggggttgtgg-3′
AQP SS9TAG-R	5′-ccacaacccccCTatactaaccaCTatgtgccaatgaattcagccac-3′
dsbAsp-AQP-F	5′-catg*ccatgg*caatgaaaaagatttggctggcgctggctggtttagttttagcgtttagcgcatcggcgatgtcagttatcacaccatatc-3′
malEsp-AQP-F	5′-catg*ccatgg*caatgaaaataaaaacaggtgcacgcatcctcgcattatccgcattaacgacgatgatgttttccgcctcggctctcgccatgtcagttatcacaccatatc-3′
ompAsp-AQP-F	5′-catg*ccatgg*caatgaaaaagacagctatcgcgattgcagtggcactggctggtttcgctaccgtagcgcaggccatgtcagttatcacaccatatc-3′
ompCsp-AQP-F	5′-catg*ccatgg*caatgaaagttaaagtactgtccctcctggtcccagctctgctggtagcaggcgcagcaaacgctatgtcagttatcacaccatatc-3′
pelBsp-AQP-F	5′-catg*ccatgg*caatgaaatacctattgcctacggcagccgctggattgttattactcgctgcccaaccagcgatggccatgtcagttatcacaccatatc-3′
phoAsp-AQP-F	5′-catg*ccatgg*caatgaaacaaagcactattgcactggcactcttaccgttactgtttacccctgtgacaaaagccatgtcagttatcacaccatatc-3′
SP-AQP-R	5′-cg*ggatcc*ttattaatgatgatgatgatgatgatcttcgtttggaca-3′

Note: mutation sites for unnatural amino acid incorporation were capitalized, the upstream signal peptide sequences and downstream His6-tag were underlined, and the restriction sites were italicized.

## Data Availability

The data used to support the findings of this study are available from the corresponding author upon request.
